# The Effects of Healthcare Quality on the Willingness to Pay More Taxes to Improve Public Healthcare: Testing Two Alternative Hypotheses from the Research Literature

**DOI:** 10.5334/aogh.2462

**Published:** 2019-11-07

**Authors:** Nazim Habibov, Rong Luo, Alena Auchynnikava

**Affiliations:** 1 School of Social Work, University of Windsor, Ontario, CA; 2 Department of Mathematic and Statistics, University of Windsor, Ontario, CA

## Abstract

The research literature discusses two opposite hypotheses regarding the possible effects of healthcare quality on the willingness to pay more taxes to improve public healthcare. One hypothesis theorizes that a lower quality of public healthcare may weaken the willingness to pay more taxes towards improving it. Another hypothesis posits that a low quality of public healthcare may strengthen the willingness to pay more taxes towards improving it. We tested both hypotheses on a diverse sample of 27 post-communist countries within Eurasia and Southern and Eastern Europe over a period of five years. We apply a binary logistic model for each country under investigation. The model is estimated by regressing the willingness to pay more taxes on six dimensions of quality, while controlling for covariates and the dummy for 2016.

We found empirical support for both hypotheses, and hence none of the hypotheses gleaned from the literature is a clear “winner.” However, we also found that the situation is less straightforward and more nuanced than is usually acknowledged within the literature. Our findings also suggest the effect is specific with respect to both a quality dimension and a country tested.

## 1. Background

The objective of this study is to explore and test two opposite theoretical hypotheses gleaned from the recent literature about effects regarding the quality of public healthcare on the willingness to pay more taxes to improve public healthcare. Does a low quality of public healthcare services weaken the willingness to pay more taxes towards improving public healthcare? Alternatively, does a low quality of public healthcare services strengthen such a willingness? Answers to the posited questions are important given the current debate regarding how much people are willing to contribute to improving the quality of public healthcare [1,2,3,4,5]. The debate is often underlined by a disjuncture between the relatively low tolerance to increased taxation for funding public healthcare, and high expectations regarding the quality of public healthcare. Indeed, research indicates both a strong rejection for increasing taxation to fund public services, and a desire for greater social spending, especially towards the support of public healthcare [6,7,8,9].

One common theoretical perspective put forth in the research literature is that a reduction in the quality of public services will weaken support for such services [10,11,12]. This perspective holds that when citizens believe that public healthcare no longer delivers a level of service that either it did in the past, or one that they expect, they will reject public healthcare as a solution, and turn instead to direct out-of-pocket expenditures or private healthcare insurance. This theoretical perspective seems to be driven by rational choice. Citizens weigh the costs and benefits related to public healthcare, and their willingness to support healthcare will be dependent on their perceptions regarding its quality. As such, a higher perceived quality of healthcare will elicit more support. On the contrary, lower perceptions regarding its quality are more likely to undermine peoples’ willingness to pay more taxes to support public healthcare. As emphasized by Andersen [13], although the “the immediate reaction to such problem [low quality of services] may be willingness to spend more, […] in the long run it may result in a decline of confidence and perhaps in a search of private alternatives. Even the most solidaristic person cannot in the long run be assumed to be willing to contribute to a system if it is considered to be inefficient.” Such an attitude inevitably results in a vicious cycle: the public healthcare system is undermined through its delivery of an unsatisfactory quality of services, thus weakening general willingness to pay taxes to support it, and so demands for private healthcare, such as for private health insurance and/or direct out-of-pocket payments, are increased. In turn, lower levels of taxation further constrain an already fiscally-pressured public healthcare system and limit its capacity to increase the quality of services, thus leading to even greater levels of dissatisfaction. Then, this cycle repeats itself. This theoretical perspective allows us to articulate the following testable hypothesis:

H_1_ Reduction in the quality of public healthcare services will weaken support for such services.

The opposite hypothesis is that a reduced quality of public services, including healthcare, will boost the willingness to pay more taxes towards improving such services [6,14,15]. As such, people may adhere to a strong normative belief that it is the government’s role to take a central place in making sure that healthcare is universally available for all. Past positive experiences with public healthcare provision may further reinforce positive beliefs about the role of government involvement in healthcare delivery. When citizens feel that public healthcare no longer delivers either the levels of services it once did, or the levels that are currently expected, the citizens will support providing more resources to public healthcare in order to improve situation. Consequently, according to this view, a low quality of public healthcare serves as an impetus for shoring up support for public healthcare. This theoretical perspective allows us to articulate the following testable hypothesis:

H_2_ Reduction in the quality of public healthcare services will strengthen support for such services.

Although both above-discussed hypotheses appear equally appealing theoretically, the specific question regarding how the quality of public healthcare services affect the willingness to pay more taxes towards improving public healthcare has rarely been the subject of empirical research. The extant literature is comprised primarily of single country studies over a single time period, and which used a single variable to capture quality. What is required however is to test these hypotheses vis-à-vis each other using a diverse sample of countries, and across time, using a rich set of quality indicators.

Against this backdrop, we test the above-discussed hypotheses for 25 post-communist countries over a five-year period using six dimensions of quality. Post-communist countries provide a rich context through which to study the effects of quality on the willingness to pay more to strengthen public healthcare since the expectations regarding healthcare, as well as its quality, have changed dramatically in these countries since the collapse of communism [5,16]. Before the collapse, these countries used the Semashko-style model which, although inefficient in many aspects, provided universal access to healthcare services that were provided free at the point of use [2]. As a result, these populations inherited expectations of universal healthcare [9]. After the collapse, contact with the West raised expectations regarding the performance of public healthcare [2]. At the same time, post-transitional processes led to the profound underfunding of public healthcare [17]. In turn, such underfunding resulted in greater official out-of-pocket expenditures and unofficial under-the-counter payments, lack of contemporary competencies, technologies, and equipment, and reductions in satisfaction with public healthcare [18,19]. Because healthcare reforms in post-communist countries will likely require additional funding [20], policy makers and healthcare planners should focus on the factors that influence the willingness to pay more to support public healthcare.

## 2. Method

### 2.1. Data and measurement

The data for this study is derived from the 2006 and 2010 rounds of the Life-in-Transition survey (LITS), which was conducted in post-communist countries by the Ipsos pollster company with funding and technical assistance from the European Bank for Reconstruction and Development and the World Bank [21,22]. The LITS covers 27 countries in three broad post-communist regions in accordance with EBRD classification [23], namely: (1) Eurasia, which encompasses Armenia, Azerbaijan, Belarus, Georgia, Kazakhstan, Kyrgyzstan, Moldova, Russia, Tajikistan, Ukraine, and Uzbekistan; (2) Southern Europe, which includes Albania, Bosnia, Bulgaria, Croatia, Macedonia, Romania, and Serbia; and (3) Eastern Europe, that covers the Czech Republic, Estonia, Hungary, Latvia, Lithuania, Poland, Slovakia, and Slovenia. The LITS uses a cross-sectional multistage design with each round of the survey and covers about 1000 respondents from each country. A detailed description of the LITS that includes a discussion of the sample design, the participation rates from each country, response rates, and socio-demographic characteristics of the sample can be found elsewhere [18,24].

Hence, we confine ourselves to a brief overview of the sampling methodology utilized in the LITS. The LITS used a multistage sampling design that consisted of five consecutive steps. In the first step, the population sample frames, which were based on the most recent census material, were obtained from the countries’ respective national statistical authorities. In the second step, these frames were employed to develop a list of Primary Selection Units (PSUs) in each country of investigation. The listed PSUs were stratified according to regions within each country and served as clusters with clearly defined borders, for instance, census enumeration areas. In the third step, using the probability proportional to size rule, between 50–70 PSUs were selected for each country for further investigation. The exact number of PSUs selected in a given country depended on: (a) the size of the country, (b) the size of the population, and (c) the density of the population. In the fourth step, after the PSUs were selected, the random walk technique was employed to identify households for interviews in each of the selected PSUs. In the fifth and final step, one member of each identified household was selected for interview with a trained interviewer based on the “last birthday” selection rule. Because of its full cross-country comparability and high quality, the LITS has been used in several comparative health studies [12,25,26].

The willingness to pay more taxes to improve public healthcare is the outcome variable of this study. The specific question asked by the LITS is “Would you be willing to give part of your income or pay more taxes, if you were sure that the extra money was used to improve the public health system?” The response to this question is in binary form (Yes = 1; No = 0). It is important to mention that this question was worded as such to emphasize that, despite the form of the contribution, it would be used towards improving public healthcare; would be available to anyone who needed it; and that this would be true not only for those who had contributed towards it (as would be the case under a private insurance mechanism or direct out-of-pocket payments). The details of the outcome variable distribution in each country are reported in Table 1.

**Table 1:** Distribution of outcome variables.

Countries	Share of respondents who are willing to pay more taxes to improve public healthcare (%)

*Panel A: Eurasia*	
Armenia	71
Azerbaijan	67
Belarus	48
Georgia	71
Kazakhstan	49
Kyrgyzstan	53
Moldova	67
Mongolia	67
Russia	38
Tajikistan	75
Ukraine	54
Uzbekistan	62
*Panel B: South Europe*	
Albania	53
Bosnia	71
Bulgaria	52
Croatia	63
Macedonia	66
Romania	42
Serbia	54
*Panel C: East Europe*	
Czech Republic	44
Estonia	46
Hungary	34
Latvia	46
Lithuania	41
Poland	34
Slovakia	29
Slovenia	45

*Note*: Data are rounded up.

The quality of public healthcare is the main predictor of interest in our study. The LITS asked respondents who had used public healthcare over the last 12 months about six dimensions of the quality of public healthcare. The six specific and separate questions asked respondents who used public healthcare whether they had experienced:

1. the frequent and unjustified absence of doctors,
2. disrespectful treatment by staff,
3. lack of availability of necessary medication,
4. long waiting times,
5. unclean facilities, and
6. the requirement of informal payments for services that are free.

The responses for all six questions are in binary form (Yes = 1; No = 0). The distribution of the predictors for each country is reported in Table 2.

**Table 2:** Distribution of predictors (%).

	Absence of doctors	Disrespectful treatment	Absence of medication	Long waiting times	Unclean facilities	Informal payments

*Panel A: Eurasia*						
Armenia	4	7	11	13	4	18
Azerbaijan	5	5	27	29	11	47
Belarus	19	32	30	70	7	13
Georgia	2	3	8	11	4	5
Kazakhstan	21	22	32	56	8	18
Kyrgyzstan	26	25	37	40	16	36
Moldova	10	16	23	30	5	28
Mongolia	7	19	24	35	6	13
Russia	13	26	26	64	6	15
Tajikistan	9	14	31	22	11	46
Ukraine	19	24	50	54	8	39
Uzbekistan	11	9	25	21	4	27
*Panel B: Southern Europe*						
Albania	12	13	24	23	17	20
Bosnia	18	25	22	49	12	16
Bulgaria	9	14	12	42	10	10
Croatia	5	15	7	47	7	4
Macedonia	24	27	35	45	32	23
Romania	5	12	28	38	13	26
Serbia	14	26	24	54	9	10
*Panel C: Eastern Europe*						
Czech Republic	3	11	6	51	3	8
Estonia	4	7	2	50	1	4
Hungary	8	13	20	57	11	4
Latvia	3	12	5	31	2	6
Lithuania	7	10	2	48	1	14
Poland	11	14	6	59	4	10
Slovakia	5	14	8	60	10	11
Slovenia	6	9	2	39	1	4

*Note*: Data are rounded up.

Following previous studies, we control for a wide range of covariates that may influence the willingness to pay more taxes for public services, including public healthcare [4,27,28]. At the individual level, we control for age, sex, marital status, education, health status, generalized trust, institutional trust, perceptions regarding the economic situation in the country, household wealth, and beliefs regarding redistribution. Descriptive statistics for the controls can be found in Table 3.

**Table 3:** Descriptive statistics for covariates.

Variable	Description	Proportion (%)	Mean	Std. Dev.

Age: 18–24	= 1 if respondents are 18–24 years old, = 0 if otherwise	9.74		
Age: 25–34	= 1 if respondents are 25–34 years old, = 0 if otherwise	18.41		
Age: 35–44	= 1 if respondents are 35–44 years old, = 0 if otherwise	17.99		
Age: 45–54	= 1 if respondents are 45–54 years old, = 0 if otherwise	17.34		
Age: 55–64	= 1 if respondents are 55–64 years old, = 0 if otherwise	16.75		
Age: 65+	= 1 if respondents are 65+ years old, = 0 if otherwise	19.76		
Female	= 1 if respondents are women, = 0 if otherwise	58.96		
Married	= 1 if respondents are married, = 0 if otherwise	58.92		
University education	= 1 if respondents have Bachelor’s degree or higher, = 0 if otherwise	20.18		
Trust people	= 1 if respondents expressed some trust or complete trust in the people, = 0 if otherwise	32.69		
Trust government	= 1 if respondents expressed some trust or complete trust into government, = 0 if otherwise	31.06		
Trust parliament	= 1 if respondents expressed some trust or complete trust into parliament, = 0 if otherwise	25.90		
Trust political parties	= 1 if respondents expressed some trust or complete trust into political parties, = 0 if otherwise	19.11		
Economic situation improved in the country	= 1 if respondents expressed agree or strongly agree that economic situation is better today than it was 4 years ago, = 0 if otherwise	26.37		
Household wealth status	The ladder of household wealth where 1 = the poorest households in the country and 10 = the richest households in the country		4.381	1.679
Believe into redistribution from the rich to the poor	= 1 if respondents agree or strongly agree that the gap between the rich and the poor should be reduced, = 0 if otherwise	77.28		
Health status	= 1 if respondents assess their health as very bad to = 5 if respondents assessed their health as very good		3.437	0.925

*Note*: Data are rounded up.

It should also be noted that some covariates are not constant, and so may vary across time. For instance, willingness to pay more taxes may change over time due to changes in the general levels of taxation in a given country. Similarly, healthcare quality is not constant and may vary due to increased or reduced public expectations or ongoing healthcare reforms. To control for time-variant characteristics, we include a dummy for the year 2016.

### 2.2. Analytic approach

Since the outcome variable is binomial, we estimate a logistic model for each country under investigation. The model is estimated by regressing the willingness to pay more taxes on six dimensions of quality, while controlling for covariates and the dummy for 2016. We use –*logit*–command in the STATA 13 statistical package.

## 3. Results

The results of logistic regression are reported in Tables 4, 5 and 6. Let us commence with Table 4, which reports results for Eurasia. In Armenia, the frequent and unjustified absence of doctors weakens the willingness to pay more for public healthcare, while lack of required medication strengthens the willingness to pay more for public healthcare. Informal payments for services that should be free bolster the willingness to support public healthcare in Azerbaijan. Disrespectful treatment by personnel strengthens willingness to support public healthcare in Kazakhstan. Three dimensions of quality are significant in Kyrgyzstan: long waiting time and unclean facilities are associated with increased support for public healthcare, whereas not having the required medication lessens such support. Long waiting time is also associated with increased support for public healthcare in Tajikistan. In Ukraine, not having the required medication is associated with an increased willingness to support public healthcare. Two quality dimensions, namely, disrespectful treatment by personnel and long waiting time, are associated with a reduced willingness to support public healthcare in Uzbekistan.

**Table 4:** Binomial logistic regression results for Eurasia (regression coefficients and standard errors in brackets).

	Armenia	Azerbaijan	Belarus	Georgia	Kazakhstan	Kyrgyzstan	Moldova	Mongolia	Russia	Tajikistan	Ukraine	Uzbekistan

Absence of doctors	–1.005*	–0.109	–0.311	0.634	–0.082	–0.214	0.220	–0.406	–0.032	–0.216	0.119	0.163
	(0.430)	(0.356)	(0.193)	(0.678)	(0.177)	(0.178)	(0.313)	(0.326)	(0.187)	(0.300)	(0.157)	(0.220)
Disrespectful treatment	–0.082	0.002	0.119	0.948	0.433*	0.254	–0.033	–0.058	–0.037	–0.344	0.112	–0.793**
	(0.342)	(0.379)	(0.166)	(0.540)	(0.179)	(0.180)	(0.238)	(0.232)	(0.152)	(0.241)	(0.148)	(0.263)
Absence of medication	0.625*	0.349	0.263	–0.204	–0.232	–0.572***	–0.262	0.318	0.031	0.089	0.316**	–0.219
	(0.286)	(0.194)	(0.163)	(0.268)	(0.152)	(0.142)	(0.207)	(0.196)	(0.153)	(0.187)	(0.115)	(0.186)
Long waiting times	0.053	0.032	–0.147	–0.036	0.166	0.700***	–0.095	0.389*	0.228	0.937***	–0.182	–0.441*
	(0.274)	(0.191)	(0.158)	(0.273)	(0.138)	(0.145)	(0.201)	(0.181)	(0.138)	(0.230)	(0.121)	(0.175)
Unclean facilities	0.609	–0.416	0.133	–0.145	–0.202	0.706***	0.168	–0.163	0.156	0.272	–0.037	–0.537
	(0.488)	(0.260)	(0.253)	(0.392)	(0.256)	(0.207)	(0.374)	(0.371)	(0.248)	(0.280)	(0.207)	(0.378)
Informal payments	–0.338	0.667***	0.309	0.015	0.114	0.151	–0.315	0.450	0.217	0.065	–0.226	0.124
	(0.210)	(0.172)	(0.214)	(0.372)	(0.183)	(0.138)	(0.198)	(0.267)	(0.176)	(0.166)	(0.120)	(0.189)
Age: 25–34	0.112	–0.016	–0.017	0.327	–0.752**	–0.405	0.048	0.007	0.002	0.075	–0.318	–0.142
	(0.348)	(0.265)	(0.255)	(0.312)	(0.256)	(0.233)	(0.415)	(0.259)	(0.252)	(0.264)	(0.238)	(0.238)
Age: 35–44	0.323	–0.154	0.049	0.660*	–0.564*	–0.354	–0.029	0.032	0.180	–0.079	–0.064	0.130
	(0.381)	(0.280)	(0.262)	(0.328)	(0.251)	(0.243)	(0.418)	(0.277)	(0.252)	(0.263)	(0.244)	(0.246)
Age: 45–54	–0.237	0.116	0.471	0.430	–0.348	–0.050	–0.234	0.426	–0.037	0.033	0.006	–0.062
	(0.358)	(0.294)	(0.277)	(0.329)	(0.259)	(0.249)	(0.406)	(0.290)	(0.265)	(0.282)	(0.249)	(0.262)
Age: 55–64	–0.718*	–0.456	–0.135	0.229	–0.553*	–0.275	–0.256	–0.132	–0.536*	–0.400	–0.257	–0.299
	(0.343)	(0.326)	(0.286)	(0.327)	(0.280)	(0.255)	(0.405)	(0.307)	(0.271)	(0.307)	(0.254)	(0.284)
Age: 65+	–1.226***	–0.391	–0.287	–0.226	–0.333	–0.408	–0.434	0.120	–0.249	0.400	–0.618*	–0.843**
	(0.341)	(0.368)	(0.324)	(0.317)	(0.306)	(0.279)	(0.407)	(0.316)	(0.286)	(0.426)	(0.254)	(0.318)
Female	–0.065	0.008	0.049	–0.184	0.212	0.092	–0.149	–0.050	0.113	0.093	–0.031	–0.158
	(0.166)	(0.163)	(0.136)	(0.158)	(0.137)	(0.129)	(0.173)	(0.154)	(0.135)	(0.160)	(0.117)	(0.138)
Married	0.101	0.429*	0.259	–0.143	0.210	–0.125	–0.008	0.034	0.083	–0.292	0.235*	0.142
	(0.174)	(0.191)	(0.144)	(0.164)	(0.137)	(0.154)	(0.187)	(0.171)	(0.130)	(0.221)	(0.117)	(0.171)
University education	0.157	–0.016	0.339*	0.395*	0.145	0.212	0.720**	–0.208	0.087	0.400	0.448***	0.010
	(0.228)	(0.183)	(0.141)	(0.173)	(0.138)	(0.153)	(0.235)	(0.170)	(0.132)	(0.216)	(0.123)	(0.192)
Trust people	–0.046	0.293	0.221	0.245	0.008	0.168	0.241	0.515**	0.380**	–0.157	0.332**	0.134
	(0.225)	(0.192)	(0.145)	(0.172)	(0.135)	(0.144)	(0.187)	(0.158)	(0.128)	(0.161)	(0.114)	(0.142)
Trust government	0.807*	0.493*	–0.010	–0.159	0.053	–0.069	–0.268	–0.101	–0.179	0.959***	0.192	–0.139
	(0.349)	(0.220)	(0.212)	(0.244)	(0.178)	(0.160)	(0.348)	(0.238)	(0.157)	(0.251)	(0.217)	(0.334)
Trust parliament	0.097	–0.066	0.165	–0.399	0.194	0.657***	0.380	–0.160	0.387*	–0.251	–0.035	0.677*
	(0.392)	(0.235)	(0.223)	(0.258)	(0.182)	(0.184)	(0.427)	(0.260)	(0.177)	(0.229)	(0.225)	(0.323)
Trust political parties	0.268	–0.012	–0.393	0.562*	0.046	–0.310	–0.288	0.581*	0.197	0.520**	0.467*	0.310
	(0.344)	(0.212)	(0.202)	(0.235)	(0.152)	(0.175)	(0.349)	(0.279)	(0.183)	(0.181)	(0.188)	(0.204)
Economic situation improved in the country	0.046	0.490**	–0.204	0.803***	0.247	–0.180	–0.021	0.232	0.342*	0.221	–0.532**	0.663**
	(0.219)	(0.167)	(0.199)	(0.191)	(0.134)	(0.158)	(0.279)	(0.209)	(0.167)	(0.183)	(0.198)	(0.207)
Household wealth status	–0.035	0.047	0.132**	0.091	0.145***	0.197***	0.179**	0.095	–0.002	–0.127*	0.114**	0.152**
	(0.047)	(0.042)	(0.047)	(0.050)	(0.043)	(0.054)	(0.057)	(0.053)	(0.032)	(0.064)	(0.039)	(0.050)
Believe into redistribution from the rich to the poor	1.098***	–0.395*	0.199	0.525**	–0.051	0.235	0.585**	0.554*	–0.276*	0.222	–0.176	0.604**
	(0.252)	(0.182)	(0.134)	(0.197)	(0.156)	(0.152)	(0.183)	(0.222)	(0.140)	(0.191)	(0.120)	(0.192)
Health statues	–0.084	–0.059	0.052	0.061	0.036	–0.141	0.029	0.023	0.123	0.171	0.028	–0.056
	(0.088)	(0.102)	(0.099)	(0.090)	(0.094)	(0.089)	(0.108)	(0.101)	(0.098)	(0.111)	(0.083)	(0.105)
2016 year	0.845***	0.715***	–0.641***	0.501**	0.688***	0.984***	0.027	0.149	–0.245	0.316	–0.471***	1.788***
	(0.199)	(0.200)	(0.155)	(0.168)	(0.144)	(0.146)	(0.173)	(0.178)	(0.135)	(0.173)	(0.119)	(0.166)
N	1038.000	964.000	1024.000	1027.000	1128.000	1181.000	907.000	849.000	1209.000	914.000	1548.000	1214.000
Log Likelihood	–527.284	–521.234	–668.994	–572.459	–741.790	–720.775	–463.297	–515.905	–766.937	–491.778	–996.000	–660.849
χ^2^	105.087	65.220	77.822	86.796	79.730	195.642	55.000	46.198	76.244	90.938	120.871	307.725
p-value	0.000	0.000	0.000	0.000	0.000	0.000	0.000	0.003	0.000	0.000	0.000	0.000

*Note*: * p < 0.05; ** p < 0.01; *** p < 0.001. Outcome variable is willingness to pay more taxes to improve public healthcare (= 1, 0 if not willing).

Moving to Table 5 with results for Southern Europe, we find that not having the necessary medication strengthens the willingness to support public healthcare in Albania, while having to make informal payments for free services lessens the willingness to support public healthcare. Long waiting time strengthens the willingness to support public healthcare in Bosnia, but weakens it in Bulgaria. In Macedonia, disrespectful treatment by personnel boosts the willingness to support public healthcare, while the lack of necessary drugs lessens the willingness to support public healthcare. Likewise, in Romania, disrespectful treatment by personnel amplifies the willingness to support public healthcare, while unclean facilities lessens the willingness to support public healthcare.

**Table 5:** Binomial logistic regression results for Southern Europe (regression coefficients and standard errors in brackets).

	Albania	Bosnia	Bulgaria	Croatia	Macedonia	Romania	Serbia

Absence of doctors	0.134	0.113	–0.036	–0.414	0.219	0.102	–0.068
	(0.218)	(0.196)	(0.253)	(0.294)	(0.182)	(0.318)	(0.185)
Disrespectful treatment	0.161	–0.288	0.379	0.086	0.529**	0.571*	0.203
	(0.217)	(0.172)	(0.200)	(0.189)	(0.176)	(0.224)	(0.143)
Absence of medication	0.344*	0.110	0.297	0.298	–0.375*	0.282	–0.104
	(0.167)	(0.179)	(0.213)	(0.248)	(0.160)	(0.182)	(0.143)
Long waiting times	–0.273	0.311*	–0.558***	–0.067	–0.164	–0.193	–0.104
	(0.173)	(0.148)	(0.141)	(0.131)	(0.149)	(0.164)	(0.124)
Unclean facilities	0.033	–0.246	0.257	–0.111	–0.191	–0.488*	–0.087
	(0.188)	(0.225)	(0.228)	(0.255)	(0.167)	(0.235)	(0.213)
Informal payments	–0.555**	–0.160	0.277	0.079	0.065	0.151	0.145
	(0.173)	(0.189)	(0.237)	(0.314)	(0.171)	(0.181)	(0.213)
Age: 25–34	–0.010	0.526*	0.628	–0.532	0.313	0.200	–0.042
	(0.268)	(0.260)	(0.347)	(0.311)	(0.251)	(0.345)	(0.296)
Age: 35–44	–0.065	0.755**	0.466	–0.350	0.253	–0.181	–0.029
	(0.283)	(0.280)	(0.337)	(0.314)	(0.265)	(0.342)	(0.298)
Age: 45–54	–0.179	0.475	0.473	–0.429	0.483	–0.080	0.121
	(0.286)	(0.282)	(0.334)	(0.317)	(0.274)	(0.361)	(0.298)
Age: 55–64	–0.346	0.341	0.047	–0.531	0.294	–0.113	–0.167
	(0.296)	(0.279)	(0.341)	(0.314)	(0.277)	(0.350)	(0.293)
Age: 65+	–0.788**	0.476	–0.271	–0.784*	0.419	–0.553	–0.372
	(0.303)	(0.282)	(0.333)	(0.312)	(0.274)	(0.342)	(0.292)
Female	–0.132	–0.020	0.110	–0.069	0.055	–0.131	–0.016
	(0.128)	(0.137)	(0.131)	(0.127)	(0.126)	(0.142)	(0.118)
Married	0.166	0.365*	0.129	0.258	0.041	0.323*	0.095
	(0.179)	(0.148)	(0.135)	(0.132)	(0.152)	(0.149)	(0.127)
University education	0.469**	0.012	0.479*	0.242	0.645**	0.625***	0.494*
	(0.154)	(0.205)	(0.187)	(0.172)	(0.200)	(0.180)	(0.208)
Trust people	–0.330*	0.465**	–0.011	0.565***	0.257	–0.373*	0.316**
	(0.145)	(0.149)	(0.160)	(0.148)	(0.156)	(0.166)	(0.121)
Trust government	–0.044	–0.714*	0.387*	0.017	0.270	0.140	–0.184
	(0.178)	(0.304)	(0.196)	(0.247)	(0.193)	(0.242)	(0.195)
Trust parliament	–0.093	0.490	–0.026	0.808**	0.102	0.283	0.558 *
	(0.193)	(0.324)	(0.280)	(0.283)	(0.209)	(0.306)	(0.222)
Trust political parties	0.081	–0.327	–0.358	0.144	–0.128	0.611	0.049
	(0.183)	(0.307)	(0.303)	(0.330)	(0.207)	(0.352)	(0.249)
Economic situation improved in the country	0.246	–0.456	0.233	0.365	–0.115	0.353	0.034
	(0.152)	(0.257)	(0.217)	(0.203)	(0.183)	(0.235)	(0.186)
Household wealth status	0.266***	0.227***	0.138**	0.308***	0.125**	0.048	0.215***
	(0.043)	(0.049)	(0.044)	(0.041)	(0.041)	(0.045)	(0.038)
Believe into redistribution from the rich to the poor	0.148	0.004	0.313	0.240	0.400*	–0.218	0.641***
	(0.153)	(0.170)	(0.164)	(0.173)	(0.175)	(0.191)	(0.175)
Health statues	–0.118	–0.003	0.056	–0.172*	0.113	0.265**	0.035
	(0.087)	(0.085)	(0.081)	(0.073)	(0.080)	(0.086)	(0.072)
2016 year	0.235	0.711***	–0.628***	–0.213	–0.125	–0.566***	–0.522***
	(0.148)	(0.141)	(0.135)	(0.133)	(0.142)	(0.166)	(0.131)
N	1136.000	1201.000	1158.000	1263.000	1274.000	973.000	1366.000
Log Likelihood	–725.438	–663.483	–720.042	–754.666	–769.826	–606.675	–868.921
χ^2^	123.941	138.121	146.789	139.260	71.349	132.841	122.801
p-value	0.000	0.000	0.000	0.000	0.000	0.000	0.000

*Note*: * p < 0.05; ** p < 0.01; *** p < 0.001. Outcome variable is willingness to pay more taxes to improve public healthcare (=1, 0 if not willing).

Finally, moving to Table 6 with results for Eastern Europe, we observe that lack of necessary medication boosts the willingness to support public healthcare in the Czech Republic. In Hungary, the frequent and unjustified absence of doctors is found to weaken support for public healthcare, while having to make informal payments strengthens support for public healthcare. In addition, long wait time and informal payments strengthen the willingness to support public healthcare in Latvia and Poland.

**Table 6:** Binomial logistic regression results for Eastern Europe (regression coefficients and standard errors in brackets).

	Czech Republic	Estonia	Hungary	Latvia	Lithuania	Poland	Slovakia	Slovenia

Absence of doctors	–0.474	0.420	–0.574*	–0.532	0.126	0.280	0.022	–0.244
	(0.334)	(0.303)	(0.259)	(0.345)	(0.230)	(0.186)	(0.286)	(0.232)
Disrespectful treatment	–0.261	0.120	0.121	0.113	0.050	–0.108	–0.074	0.287
	(0.187)	(0.226)	(0.198)	(0.170)	(0.187)	(0.169)	(0.192)	(0.199)
Absence of medication	0.673**	0.041	–0.211	–0.102	0.520	0.166	–0.189	–0.104
	(0.251)	(0.446)	(0.174)	(0.269)	(0.375)	(0.235)	(0.242)	(0.399)
Long waiting times	0.128	–0.127	–0.037	0.306**	–0.045	0.163	–0.039	–0.150
	(0.117)	(0.117)	(0.130)	(0.118)	(0.110)	(0.113)	(0.136)	(0.111)
Unclean facilities	–0.590	0.905	–0.150	0.335	0.894	–0.412	–0.064	–0.117
	(0.356)	(0.738)	(0.219)	(0.388)	(0.734)	(0.290)	(0.218)	(0.485)
Informal payments	–0.091	–0.495	0.652*	–0.388	0.024	0.377*	0.193	–0.346
	(0.213)	(0.289)	(0.312)	(0.219)	(0.160)	(0.182)	(0.203)	(0.279)
Age: 25–34	–0.235	0.032	0.240	–0.127	–0.111	–0.149	0.089	–0.024
	(0.267)	(0.288)	(0.314)	(0.237)	(0.242)	(0.258)	(0.272)	(0.243)
Age: 35–44	–0.299	–0.413	0.131	–0.362	–0.283	–0.010	0.013	–0.151
	(0.268)	(0.286)	(0.316)	(0.243)	(0.250)	(0.257)	(0.274)	(0.246)
Age: 45–54	–0.371	–0.419	–0.136	0.049	0.127	–0.334	0.082	–0.209
	(0.276)	(0.286)	(0.321)	(0.240)	(0.243)	(0.268)	(0.284)	(0.249)
Age: 55–64	–0.392	–0.635*	0.362	–0.313	–0.077	–0.300	0.004	–0.091
	(0.281)	(0.286)	(0.305)	(0.244)	(0.247)	(0.262)	(0.298)	(0.251)
Age: 65+	–0.542	–0.983***	–0.254	–1.007***	–0.463	–0.488	0.038	–0.224
	(0.279)	(0.277)	(0.303)	(0.235)	(0.243)	(0.267)	(0.308)	(0.245)
Female	0.093	0.046	0.121	0.193	–0.138	–0.025	0.059	0.179
	(0.115)	(0.123)	(0.127)	(0.110)	(0.112)	(0.108)	(0.130)	(0.105)
Married	0.171	0.269*	0.193	0.223*	0.233*	–0.039	0.124	0.197
	(0.121)	(0.118)	(0.129)	(0.109)	(0.114)	(0.115)	(0.137)	(0.113)
University education	0.441*	0.073	0.039	0.085	0.393***	0.226	0.156	0.121
	(0.202)	(0.142)	(0.187)	(0.133)	(0.118)	(0.162)	(0.212)	(0.151)
Trust people	0.124	–0.127	0.058	0.272*	0.067	–0.151	–0.070	0.222
	(0.134)	(0.116)	(0.142)	(0.116)	(0.114)	(0.114)	(0.153)	(0.119)
Trust government	–0.144	0.219	0.491*	0.132	0.031	0.681***	–0.067	0.207
	(0.186)	(0.143)	(0.192)	(0.209)	(0.145)	(0.163)	(0.230)	(0.218)
Trust parliament	0.044	0.416**	–0.190	0.045	0.448*	–0.079	0.250	–0.026
	(0.215)	(0.156)	(0.210)	(0.226)	(0.199)	(0.177)	(0.258)	(0.258)
Trust political parties	0.000	0.085	–0.039	0.170	–0.039	–0.030	0.128	–0.040
	(0.231)	(0.161)	(0.223)	(0.262)	(0.204)	(0.176)	(0.252)	(0.256)
Economic situation improved in the country	0.080	0.162	0.083	0.275	0.186	0.316*	0.101	0.638***
	(0.136)	(0.143)	(0.172)	(0.146)	(0.140)	(0.127)	(0.162)	(0.187)
Household wealth status	0.171***	0.117**	0.186***	0.100**	0.088*	0.105**	0.033	0.179***
	(0.046)	(0.040)	(0.045)	(0.037)	(0.038)	(0.035)	(0.042)	(0.037)
Believe into redistribution from the rich to the poor	0.043	0.659***	–0.220	0.540***	–0.049	–0.005	0.027	0.408*
	(0.125)	(0.171)	(0.180)	(0.160)	(0.173)	(0.121)	(0.184)	(0.168)
Health statues	0.078	0.063	0.036	0.141	0.287***	0.031	0.299***	–0.004
	(0.075)	(0.079)	(0.080)	(0.076)	(0.075)	(0.071)	(0.088)	(0.066)
2016 year	0.203	0.343**	–0.023	–0.307**	0.259*	–0.434***	–0.886***	–0.168
	(0.129)	(0.133)	(0.138)	(0.117)	(0.125)	(0.111)	(0.141)	(0.115)
N	1370.000	1402.000	1264.000	1674.000	1638.000	1648.000	1344.000	1588.000
Log Likelihood	–914.224	–906.743	–768.303	–1074.938	–1054.626	–1041.064	–773.105	–1054.135
χ^2^	62.253	129.812	74.803	167.145	132.932	111.231	97.146	78.207
p-value	0.000	0.000	0.000	0.000	0.000	0.000	0.000	0.000

*Note*: * p < 0.05; ** p < 0.01; *** p < 0.001. Outcome variable is willingness to pay more taxes to improve public healthcare (= 1, 0 if not willing).

## 4. Discussion

The results presented above lead to broad and rather complex interpretations since most of the significant effects can be attributed, to various degrees, to both tested hypotheses depending on the country under investigation, and the dimension of quality being discussed. In order to reduce this complexity and facilitate a discussion of the findings, the results are summarized in Table 7 and are discussed as follows:

**Table 7:** Support for the tested hypotheses by countries and quality dimensions.

Health quality	Support for Hypothesis 1: Reduction in the quality of public healthcare services will weaken support for such services	Support for Hypothesis 2: Reduction in the quality of public healthcare services will strengthen support for such services

Absence of doctors	Armenia, Hungary	None
Disrespectful treatment	Uzbekistan	Kazakhstan, Macedonia, Romania
Absence of medication	Kyrgyzstan, Macedonia	Armenia, Ukraine, Albania, Czech Republic
Long waiting times	Uzbekistan, Bulgaria	Kyrgyzstan, Tajikistan, Bosnia, Latvia
Unclean facilities	Romania	Kyrgyzstan
Informal payments	Albania	Azerbaijan, Hungary, Poland

First, country-wise, we found support for Hypothesis 1, which postulates that a lower quality of public healthcare will weaken the willingness to pay more taxes in order to improve it in Armenia, Kyrgyzstan, Uzbekistan, Albania, Bulgaria, Macedonia, Romania, and Hungary. Regarding quality dimensions, Hypothesis 1 is confirmed with respect to frequent and unjustified absence of doctors, absence of required medication, disrespectful treatment by personnel, long waiting time, unclean facilities, and payments required for services that should be free.

Second, with regard to the six quality dimension, we found support for Hypothesis 2, which posits that a low quality of public healthcare will strengthen the willingness to pay more taxes towards its improvement in Armenia, Azerbaijan, Kazakhstan, Kyrgyzstan, Tajikistan, Ukraine, Albania, Bosnia, Macedonia, Romania, Czech Republic, Hungary, Latvia, and Poland. From the perspective of quality dimensions, Hypothesis 1 is confirmed for disrespectful treatment by personnel, absence of required medication, long waiting time, unclean facilities, and payments required for services that should be free.

Third, in some countries both Hypothesis 1 and Hypothesis 2 are true depending on the dimension of quality tested. These countries are Armenia, Kyrgyzstan, Uzbekistan, Albania, Macedonia, Romania, and Hungary. For instance, in Hungary the frequent and unjustified absence of doctors weakens the willingness to pay more to improve public healthcare, while unofficial payment has an opposite effect. For all other countries, only one of the tested hypotheses was found to be true.

Finally, the frequent and unjustified absence of doctors is the only quality dimension that always weakens the willingness to pay more taxes towards its improvement. All other quality dimensions have varying effects depending on the country. For instance, long waiting time weakened the willingness to pay more taxes towards improving public healthcare in Bulgaria and Uzbekistan, but strengthened it in Bosnia, Latvia, Kyrgyzstan, and Tajikistan.

## 5. Limitations

We need to acknowledge the limitations of this study. To begin, because of the cross-sectional nature of our data, we are not able to make any assertions regarding causality. Second, our data set contains only information about the quality of healthcare from the perspective of patients. Other ways to measure quality include using publicly available data from national quality information systems, measuring the opinions of key informants and healthcare professionals, and soliciting the perceptions of the population at large [29,30]. Third, the existing literature lacks a theoretical framework regarding the effect of patients’ perspectives on the quality of public healthcare and also lack agreement regarding which quality indicators should be analysed [5,8]. That being said, examining patients’ perspectives on the quality of healthcare can be seen as empowering patients, politicians, and healthcare planners with the knowledge required to make informed decisions about healthcare reforms and payment strategies. Furthermore, it can also be viewed as cultivating the accountability and transparency of public healthcare [31]. Additionally, because patients are the ones who experience healthcare directly, their views on the quality of the provision of healthcare may bring issues to the surface that have not been revealed through other methods. One such example is the question of how healthcare reforms filter down to the level that is experienced by patients, and to what extent the reforms are acceptable to patients [32,33,34].

## 6. Conclusion and direction of future studies

In this study, we focus on the effect of the quality of public healthcare on the willingness to pay more taxes towards improving healthcare. The current literature is not consistent in explaining the direction of this effect. Two opposite hypotheses are frequently put forth: one that suggests that a lower quality of public healthcare will strengthen the willingness to pay more taxes towards improving it, and the opposite hypothesis by which a lower quality of public healthcare is thought to weaken the willingness to pay more taxes towards improving it.

In light of the above, this is the first study that has tested both hypotheses in the field of healthcare services. As far as we are aware, previous studies were conducted in the areas of education and social welfare, and the studies that have been conducted in the field of healthcare have focused on the analysis of general satisfaction with healthcare and not on its specific dimensions of quality. This is also the first study to focus on a large sample of diverse post-communist countries in Eurasia, and Southern and Eastern Europe over a five-year period.

The main theoretical interpretation of our findings is that a “one size fits all” approach cannot be assumed by healthcare planners and administrators. Rather, there exists considerable variation across countries in terms of the effects of various quality dimensions on the willingness to support public healthcare. This should be expected, inasmuch as we found empirical support for both tested hypotheses. As such, none of the hypotheses gleaned from the literature is a clear “winner.” However, we also found that the situation is less straightforward and more nuanced than the extant literature acknowledges. In fact, our findings suggest that the effect is *quality dimension-specific*. Thus, within the same country, some dimensions of quality have a positive effect on the willingness to pay more taxes towards improving public healthcare, while other dimensions have an opposite negative effect. Similarly, our findings suggest that the effect of quality is *country-specific*. Thus, the same quality indicator may have a positive effect on the willingness to pay more taxes towards improving healthcare in some countries, and a negative effect in other countries.

Our main practical interpretation is that for each country under investigation, we provide information regarding which dimension of quality should be addressed to increase support for public healthcare. Let us consider Kyrgyzstan as an illustration of this point. Long waiting times and unclean facilities in Kyrgyzstan strengthen the willingness to pay more taxes to improve public healthcare. In contrast, the absence of medication weakens the willingness to pay more taxes to support public healthcare, while the absence of doctors, disrespectful treatment by healthcare personnel, and informal payments have no significant effect. Consequently, making a clear link between the amounts of extra taxes that will be collected towards increasing the availability of medication will likely increase the willingness to support public healthcare reforms in Kyrgyzstan. In contrast, emphasizing improvements with respect to the absence of doctors, disrespectful treatment by healthcare personnel, and the need for informal payments is unlikely to generate considerable support for healthcare reform. Similar types of interpretations could be made for each country under investigation.

In contrast to other studies that have focused solely on satisfaction with healthcare, our analysis also provides new insights into cross-country differences. For example, patients in Hungary report comparatively lower levels of dissatisfaction with services than do those in other countries surveyed since the Hungarian healthcare system ranks highest in the Euro Health Consumer Index [35]. In addition, the relatively high level of satisfaction expressed in Hungary has also been reported in previous studies [5]. In contrast, our study found that the absence of doctors as well as informal payments were significant precursors to support for public healthcare in that country. Similarly, the previous studies which focused on healthcare satisfaction within the countries of the former Soviet Union reported the highest levels of satisfaction in Azerbaijan and Armenia and the lowest in Moldova [2]. In contrast, our study reveals that respondents stress the negative effects of the absence of doctors and medication in Armenia and informal payments in Azerbaijan. Furthermore, this particular result is consistent with the finding that Azerbaijan is at the lead of countries with respect to the negative impact of informal payments on healthcare satisfaction [12].

As such, the results of our study are also in line with those of previous studies on healthcare satisfaction that have reported only minimal agreement across countries [2,5,28]. The inconsistency across countries may be the result of variations in culture, political context, and the influence of the mass-media [8,36]. Furthermore, inconsistencies could also be rooted in variations in expectations regarding healthcare services, especially when a single relatively generic indicator such as satisfaction with healthcare is employed [37,38]. Moreover, the existence of this type of discrepancy could also signify weaknesses with respect to the indicators of satisfaction and quality used in this and previous studies [39,40].

In light of the evidence presented and discussed in this study, future studies should focus on several issues. First, it would be valuable to develop a framework with respect to healthcare quality. Developing such a framework would help identify a set of quality indicators that could be tested for their validity in cross-country and cross-time studies. Second, there is a need to move beyond the analysis of general satisfaction with public healthcare. Instead, efforts should be channelled towards examining the fine details of healthcare performance, including its quality and effectiveness. Finally, future studies should combine different dimensions of data such as the opinions of key informants, the perceptions of patients, healthcare providers, and the general public, as well as the incorporation of government statistics.

## Data Availability

The datasets generated and/or analysed during the current study are available in the European Bank for Reconstruction and Development (EBRD), Life in transition country repository.

## Abbreviations

LITS: Life in Transition survey; EBRD: European Bank for Reconstruction and Development.
